# The role of high-fat diets in cancer mortality: is butter to blame?

**DOI:** 10.1097/MS9.0000000000003544

**Published:** 2025-07-18

**Authors:** Aly Omer Patel, Hafsa Ali, Mohammed Mahmmoud Fadelallah Eljack

**Affiliations:** a United Medical and Dental College, Karachi, Pakistan; b Sindh Medical College, Jinnah Sindh Medical University, Karachi, Pakistan; c Community Department, University of Bakht Alruda, Ad Duwaym, Sudan

Dietary fat intake has been a topic of interest for researchers due to its significant association in the pathogenesis of multiple conditions, such as cardiovascular disorders, diabetes, and cancer. High-fat foods such as butter change gut microbiota, contributing to the activation of the inflammatory pathway along with promoting obesity, both of which exacerbate carcinogenesis.^[1]^

A study by Zhang et al. explored the association between high butter consumption and increased risk of dying from cancer. The analysis is timely because the role of different dietary fats requires closer attention as we move toward constantly changing dietary habits. Assessing the relationship of consumption of butter and oil with mortality following a 33-year follow-up of more than 200,000 adults in three large US cohorts, the study observed that higher intakes of plant oils such as olive, canola, and soybean were associated with a 16% lower mortality; higher butter consumption was associated with a 15% greater risk of total mortality. A 10-gram per day increase in butter consumption was associated with a 12% greater risk of cancer mortality; replacement with vegetable oils resulted in a 17% reduction in both cancer and cardiovascular mortality^[2]^. These findings are further supported by a meta-analysis of prospective cohort studies, which reports that a diet high in saturated fat is correlated to a 4% increase in cancer mortality for every 5% increase in energy from saturated fats. On the other hand, a diet with a high content of polyunsaturated fats.^[3]^

The distinctive lipid compositions of butter, relative to plant oils, may be among the underlying biological processes explaining these effects. With saturated fatty acid composition, butter is associated with rises in low-density lipoprotein (LDL) cholesterol and promotion of inflammation, factors responsible for chronic disease risk, as identified by Mensink et al^[4]^. Adding on, the increase in reactive oxygen species, nitrogen species, hypertrophied fat cells, and macrophages all cause an exhaustion of the antioxidant system of the cell, inducing oxidative stress, which aggravates inflammation. This chronic inflammation is highly responsible for cancer pathogenesis.^[1]^ The unsaturated fats in plant oils, on the other hand, are beneficial to lipid profiles as well as having anti-inflammatory effects, conferring protection against cancer and cardiovascular disease^[5]^. However, caution should be exercised not to overemphasize the potential limitations. The imposition of measurement error by employing a self-reported diet brings error; the largely White composition of healthcare staff might limit the generalizability of results. Previous research has suggested that dietary fat-cancer risk interaction is complicated and depends on several other diet and lifestyle variables.

In a 2014 meta-analysis by Schwingshackl and Hoffmann, the substitution of polyunsaturated with saturated fats reduced cardiovascular mortality risk; evidence for cancer mortality is not as strong^[6]^. To validate such findings, follow-up studies need to examine heterogeneous groups and adjust for disease-specific results.

However, it is important to approach these findings with an understanding of their limitations; the reliance on self-reported dietary data can lead to bias. Moreover, the relation of dietary fat to cancer is complex and multifactorial due to the contribution of overall dietary patterns and physical activity, which can also lead to cancer, in addition to fat intake.^[7]^ Another factor that can influence the results is the socioeconomic status of an individual, as observed in 2012, increased body fat due to saturated fat consumption accounted for 4% of cancers worldwide and 7-8% in high-income countries.^[1]^

Based on these results, public health guidelines should still emphasize the importance of replacing saturated fat with unsaturated fats to offset disease risk. Instead of focusing only on individual nutrients, a holistic approach should be adopted, promoting whole-diet changes to encourage consumption of plant-based oils, providing healthy recipes to incorporate these oils, accounting for cultural preferences, and encouraging frequent visits to a nutritionist can all help promote a low saturated fat diet. The results vindicate the role of food choice in preventing chronic diseases and emphasize the benefits of substituting vegetable oils for animal fats to attain healthier alternatives.

## Data Availability

Data sharing does not apply to this article as no new data were created or analyzed in this study.

## References

[1] Bojková B Winklewski PJ Wszedybyl-Winklewska M. Dietary fat and cancer-which is good, which is bad, and the body of evidence. Int J Mol Sci 2020;21(11):4114. 32526973 10.3390/ijms21114114 PMC7312362

[2] Zhang Y Chadaideh KS Li Y. Butter and plant-based oils intake and mortality. JAMA Internal Medicine, Mar 2025;185:549. 10.1001/jamainternmed.2025.0205 PMC11886867 40048719

[3] Kim Y Je Y Giovannucci EL. Association between dietary fat intake and mortality from all-causes, cardiovascular disease, and cancer: a systematic review and meta-analysis of prospective cohort studies. Clin Nutr 2021;40:1060–70. 32723506 10.1016/j.clnu.2020.07.007

[4] Mensink R. Effects of the individual saturated fatty acids on serum lipids and lipoprotein concentrations. Am J Clin Nutr. 1993;57:711S–714S. 8475888 10.1093/ajcn/57.5.711S

[5] Hooper L Summerbell CD Thompson R. Reduced or modified dietary fat for preventing cardiovascular disease. Sao Paulo Med J 2016;134(2):182–83. 27224282 10.1590/1516-3180.20161342T1 PMC10496532

[6] Schwingshackl L, and Hoffmann G. Monounsaturated fatty acids, olive oil and health status: a systematic review and meta-analysis of cohort studies. Lipids Health Dis 2014;13(1):154 25274026 10.1186/1476-511X-13-154 PMC4198773

[7] Kruk J Marchlewicz M. Dietary fat and physical activity in relation to breast cancer among Polish women. Asian Pac J: APJCP 2013;14(4):2495–502. 10.7314/apjcp.2013.14.4.2495 23725163

